# Caffeic Acid Phenethyl Ester Suppresses Proliferation and Survival of TW2.6 Human Oral Cancer Cells via Inhibition of Akt Signaling

**DOI:** 10.3390/ijms14058801

**Published:** 2013-04-24

**Authors:** Ying-Yu Kuo, Hui-Ping Lin, Chieh Huo, Liang-Cheng Su, Jonathan Yang, Ping-Hsuan Hsiao, Hung-Che Chiang, Chi-Jung Chung, Horng-Dar Wang, Jang-Yang Chang, Ya-Wen Chen, Chih-Pin Chuu

**Affiliations:** 1 Institute of Cellular and System Medicine, National Health Research Institutes, Miaoli 35053, Taiwan; E-Mails: jennykuo0101@yahoo.com.tw (Y.-Y.K.); diablofish@nhri.org.tw (H.-P.L.); jason429w@yahoo.com.tw (C.H.); liangcheng610@nhri.org.tw (L.-C.S.); jtyang08@gmail.com (J.Y.); s9732010@mail.nchu.edu.tw (P.-H.H.); 2 Translational Center for Glandular Malignancies, National Health Research Institutes, Miaoli 35053, Taiwan; E-Mail: jychang@nhri.org.tw; 3 Department of Life Sciences, National Central University, Taoyuan City 32001, Taiwan; 4 Institute of Biotechnology, National Tsing Hua University, Hsinchu City 30013, Taiwan; E-Mail: hdwang@life.nthu.edu.tw; 5 Division of Environmental Health and Occupational Medicine, National Health Research Institutes, Miaoli 35053, Taiwan; E-Mail: hcchiang@nhri.org.tw; 6 National Center for Toxicological Research, National Health Research Institutes, Miaoli 35053, Taiwan; 7 Department of Health Risk Management, China Medical University, Taichung City 40402, Taiwan; E-Mail: cjchung@mail.cmu.edu.tw; 8 National Institute of Cancer Research, National Health Research Institutes, Miaoli 35053, Taiwan; E-Mail: ywc@nhri.org.tw; 9 Graduate Program for Aging, China Medical University, Taichung City 40402, Taiwan; 10 Ph.D. Program in Tissue Engineering and Regenerative Medicine, National Chung Hsing University, Taichung City 40227, Taiwan

**Keywords:** oral cancer, caffeic acid phenethyl ester, TW2.6, cell proliferation, cell cycle, Akt, Akt1, Akt2, phospho-Akt Ser473, phospho-Akt Thr 308, FOXO1, FOXO3a, phospho-FOXO1 Thr24, phospho-FoxO3a Thr32, NF-κB, phospho-NF-κB Ser536, Rb, phospho-Rb Ser807/811, Skp2, cyclin D1, p27, 5-fluorouracil

## Abstract

Caffeic acid phenethyl ester (CAPE) is a bioactive component extracted from honeybee hive propolis. Our observations indicated that CAPE treatment suppressed cell proliferation and colony formation of TW2.6 human oral squamous cell carcinoma (OSCC) cells dose-dependently. CAPE treatment decreased G1 phase cell population, increased G2/M phase cell population, and induced apoptosis in TW2.6 cells. Treatment with CAPE decreased protein abundance of Akt, Akt1, Akt2, Akt3, phospho-Akt Ser473, phospho-Akt Thr 308, GSK3β, FOXO1, FOXO3a, phospho-FOXO1 Thr24, phospho-FoxO3a Thr32, NF-κB, phospho-NF-κB Ser536, Rb, phospho-Rb Ser807/811, Skp2, and cyclin D1, but increased cell cycle inhibitor p27^Kip^. Overexpression of Akt1 or Akt2 in TW2.6 cells rescued growth inhibition caused by CAPE treatment. Co-treating TW2.6 cells with CAPE and 5-fluorouracil, a commonly used chemotherapeutic drug for oral cancers, exhibited additive cell proliferation inhibition. Our study suggested that administration of CAPE is a potential adjuvant therapy for patients with OSCC oral cancer.

## 1. Introduction

Head and neck cancers rank the 6th most common cancers worldwide, affecting 650,000 people and causing 350,000 deaths per year [1,2]. Oral cancer is the most frequent cancer of head and neck cancers. There are several types of oral cancers. More than 90% of oral cancers are oral and oropharyngeal squamous cell carcinoma (OSCC) [2,3]. There were approximately 400,000 new cases and 200,000 deaths of OSCC worldwide in 2008 (http://www-dep.iarc.fr/) [3]. Forty thousand OSCC cases were diagnosed and 8,000 patients died from OSCC in the United States in 2012 [3,4]. The overall 5-year survival rate of OSCC patients is approximately 60% [4]. The poor prognosis of OSCC is due to the low response rate to current therapeutic drugs [2]. Environmental carcinogens, such as betel quid chewing, tobacco smoking, and alcohol drinking, have been identified as major risk factors for head and neck cancers [5]. The incidence of oral cancer is highest in Southeast Asia and central African countries [6]. According to the statistics of Taiwanese Department of Health, oral cancer ranks the 4th most common cancer in male in Taiwan in 2011. Oral cancer is the fastest growing malignancies in Taiwan. The majority of the oral cancer patients in Taiwan are regular users of betel quid [5]. Betal quid is a combination of betel leaf, areca nut, and slaked lime [5]. The cumulative effects of betel quid chewing, alcohol drinking, and tobacco smoking increase 123-fold in risk of oral cancer in Taiwanese patients [5]. TW2.6 is an OSCC cancer cell line established from the untreated primary squamous cell carcinoma of the buccal mucosa from a 48-year-old betel quid chewing and tobacco smoking Taiwanese male patient [7]. TW2.6 cells have morphological features of keratinocytes with a doubling time of 24 h [7]. TW2.6 is a useful cell line model for investigating drug response of OSCC cancer cells.

Caffeic acid phenethyl ester (CAPE) is a strong antioxidant extracted from honeybee hive propolis and [8,9]. CAPE is a well known NF-κB inhibitor at high concentrations (50–80 μM) [9]. CAPE treatment suppresses proliferation of several human cancer cell lines, including breast [10,11], prostate [12–15], lung [16,17], and cervical [18] cancer cells. CAPE treatment has also been reported to suppress human oral cancer cells. CAPE treatment causes G2/M arrest in Japanese squamous cell carcinoma SAS cells and Taiwanese oral epidermoid carcinoma OEC-M1 cells (IC_50_ 130 μM and 160 μM, respectively) [19]. CAPE treatment does not affect proliferation of normal human oral fibroblast (NHOF) cells at concentration lower than 100 μM [19], suggesting that CAPE exhibits selective suppressive effect on human oral cancer cells. However, the molecular mechanism lying underneath is not understood. Our recent studies suggested that CAPE treatment causes growth inhibition and G1 cell cycle arrest in human prostate cancer cells by suppressing Akt signaling [12–15]. We thus examine the effect of CAPE treatment on cell proliferation, cell cycle, and signaling protein expression in TW2.6 human oral cancer cells.

## 2. Results

### 2.1. CAPE Treatment Suppressed the Proliferation and Survival of TW2.6 Human Oral Cancer Cells

**Figure 1:**
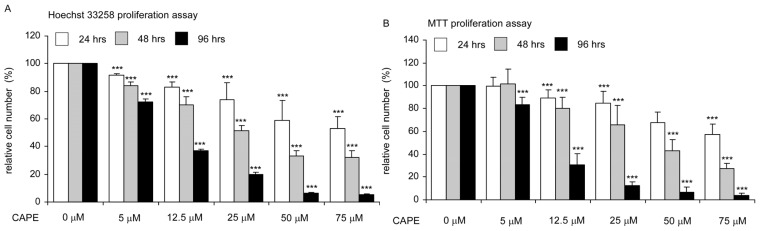
Effect of caffeic acid phenethyl ester (CAPE) on viability and proliferation of TW2.6 oral cancer cells. TW2.6 oral cancer cells were treated with increasing concentrations of CAPE for 24, 48, or 96 h and determined by Hoechst dye 33258-based 96-well proliferation assay (**A**) or by MTT (3,4,5-dimethylthiazol-2-yl)-2-5-diphenyltetrazolium bromide) 96-well assay (**B**), respectively, as described in Material and Methods. Cell numbers were normalized to control (DMSO treatment) of each treatment period. The mean and standard deviation represented the average and standard deviation respectively of the results from all 36 wells in the three experiments. Asterisk *** represents statistically significant difference *p* < 0.001 between the treated group and the control group.

Hoechst dye-based proliferation assay indicated that CAPE treatment suppressed the proliferation of TW2.6 human OSCC cancer cells (Figure 1A). The inhibitory effect was dose-dependently and accumulated over time. Hoechst dye 33285 proliferation assays indicated that the IC_50_ of CAPE to suppress proliferation of TW2.6 cells was 72.1 μM, 41.5 μM, and 19.0 μM for 24, 48, and 96 h treatment, respectively (Figure 1A). MTT assay suggested that CAPE treatment decreased survived cells. The IC_50_ of CAPE to suppress survival of TW2.6 cells determined by MTT assays was 83.8 μM, 46.6 μM, and 18.8 μM for 24, 48, and 96 h treatment, respectively (Figure 1B). The IC_50_ detected by MTT assay was very similar to the IC_50_ detected by Hoechst dye-based proliferation assay, suggesting that inhibition of cell proliferation was responsible for the reduction of viable cells caused by CAPE treatment in TW2.6 oral cancer cells.

### 2.2. CAPE Treatment Suppressed TW2.6 Cells Soft Agar Colony Formation and NF-κB Activity in TW2.6 Cells

**Figure 2:**
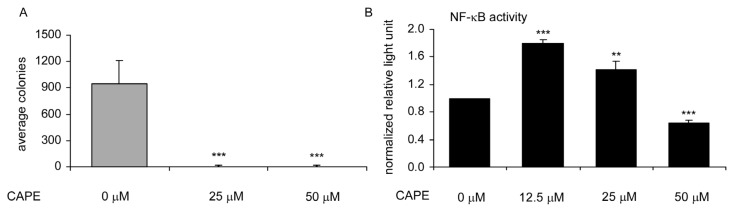
Effects of CAPE treatment on soft agar colony formation and NF-κB activity of TW2.6 cells. (**A**) TW2.6 cells were treated with 0, 25, or 50 μM CAPE for 16 days. Asterisk (***) represents statistically significant difference (*p* < 0.001) between the treated group and the control group; (**B**) TW2.6 cells were transfected with pRL-TK-Renilla luciferase plasmid and 4X NF-κB reporter gene vector. Twenty four hours after transfection, cells were treated with 0, 12.5, 25, and 50 μM of CAPE. After an additional 24 h, cells were lysed in 100 μL passive lysis buffer and luciferase activity was measured using a Dual-Luciferase kit (Promega) in a 20/20^n^ luminometer Turner Biosystems. Experiments were repeated three times. Error bars represented standard deviation.

Soft agar colony formation assay revealed that treatment with 25 μM or 50 μM CAPE totally blocked the formation of TW2.6 colonies in soft agar, confirming the anti-cancer activity of CAPE against TW2.6 oral cancer cells (Figure 2A). Since CAPE was previously reported as an NF-κB inhibitor [9,12,13], we determined whether CAPE can inhibit NF-κB activity in TW2.6 cells using a plasmid-based luciferase reporter assay. CAPE treatment at 12.5 μM or 25 μM increased NF-κB activity, while CAPE treatment at 50 μM suppressed NF-κB activity (Figure 2B). Reduction of NF-κB activity by CAPE treatment at high concentration may partially contribute to growth inhibition of TW2.6 cells.

### 2.3. CAPE Treatment Caused Dysregulation of Cell Cycle

**Figure 3:**
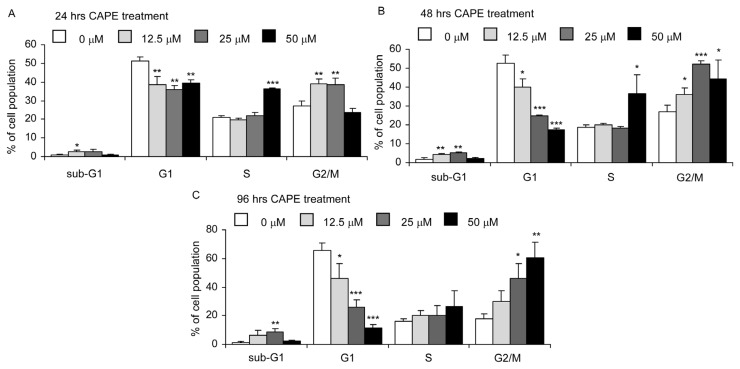
Effects of CAPE on cell cycle distribution of TW2.6 oral cancer cells. TW2.6 oral cancer cells were treated with 0, 12.5, 25, and 50 μM CAPE for 24 h (**A**), 48 h (**B**), and 96 h (**C**). Cell cycle distribution was determined by flow cytometry. Asterisk (*, **, ***) represents statistically significant difference (*p* < 0.05, *p* < 0.01 and *p* < 0.001, respectively) between the treated group and the control group.

We next performed flow cytometric analysis to determine if cell cycle progression of TW2.6 oral cancer cells is affected by CAPE. Treatment with increasing concentration (0, 12.5, 25, 50 μM) of CAPE for 24, 48, and 96 h caused a decrease of G1 phase cell population, an increase of S phase cell population at high dosage (50 μM), and an increase of G2/M cell population (Figure 3A–C). The effects were more significant at 48 and 96 h treatment compared to 24 h treatment. These observations indicated that CAPE may induce G2/M arrest in TW2.6 cells. Treatment with 12.5–25 μM CAPE slightly increased sub-G1 population in TW2.6 cells.

### 2.4. CAPE Treatment Induced Apoptosis in TW2.6 Cells

**Figure 4:**
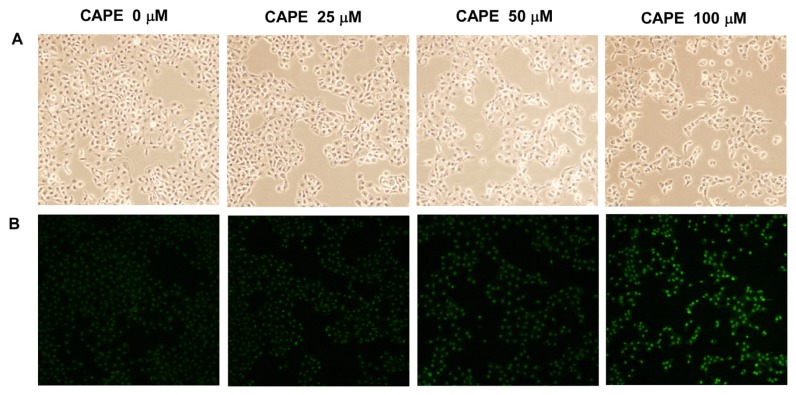
Treatment of high concentration of CAPE induced apoptosis in TW2.6 oral cancer cells. TW2.6 oral cancer cells were treated with 0, 25, 50, and 100 μM CAPE for 48 h. Cell morphology was determined by light microscopy (**A**). TUNEL assay was performed as described in Material and Methods to determine the apoptotic cell population (**B**). Green fluorescent light indicated apoptotic cells stained with TUNEL. Images were viewed at 200X with Olympus confocal microscope.

As PI staining flow cytometry analysis was not a reliable method to detect apoptosis, we introduced TUNEL assay to determine if CAPE treatment at higher concentrations may induce apoptosis in TW2.6 oral cancer cells. We treated TW2.6 cells with 0, 25, 50 and 100 μM CAPE for 48 h. Treatment with 100 μM CAPE for 48 h significantly reduced cell numbers (Figure 4A) and induced apoptosis in TW2.6 cancer cells (Figure 4B).

### 2.5. CAPE Caused a Reduction in Abundance of Signaling Proteins Regulating Cell Cycle and Akt Activity

**Figure 5:**
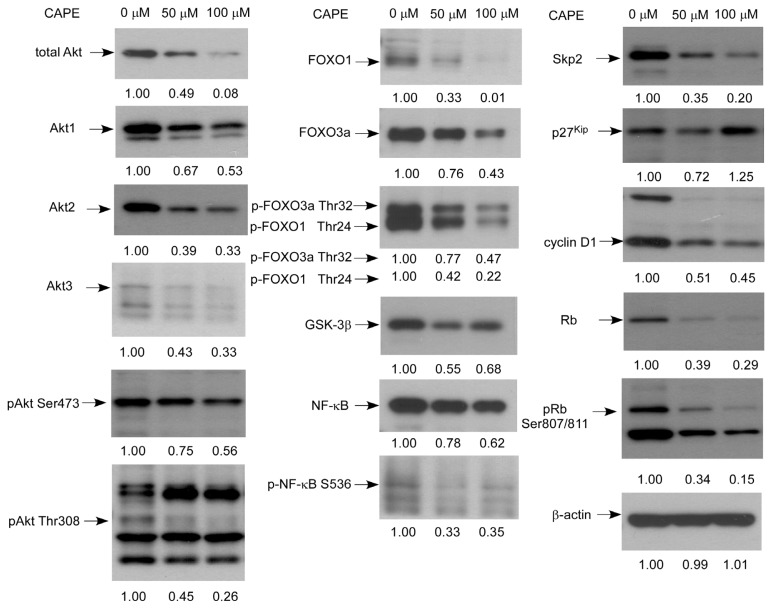
Effects of CAPE treatment on the abundance and phosphorylation status of signaling proteins. Protein expression of total Akt, Akt1, Akt2, Akt3, phospho-Akt Ser473, phospho-Akt Thr308, GSK3β, Rb, phospho-Rb Ser807/811, cyclin D1, and Skp2, FOXO1, FOXO3a, phospho-FOXO1 Thr24, phospho-FOXO3a Thr32, NF-κB, phospho-NF-κB Ser536, p27^Kip^, and β-actin in TW2.6 cells treated with 0, 50, or 100 μM CAPE for 48 h were assayed by Western blotting. Experiments were repeated three times.

CAPE treatment caused a decrease in protein expression level of total Akt, Akt1, Akt2, Akt3, phospho-Akt Ser473, phospho-Akt Thr308, GSK3β, retinoblastoma protein (Rb), phospho-Rb Ser807/811, cyclin D1, and S-phase kinase-associated protein 2 (Skp2), forkhead box protein O1 (FOXO1), FOXO3a, phospho-FOXO1 Thr24, phospho-FOXO3a Thr32, NF-κB, phospho-NF-κB Ser536, but increased the protein abundance of cell cycle inhibitor p27^Kip^ in TW2.6 cells (Figure 5).

### 2.6. Overexpression of Akt1 or Akt2 Rescued Growth Inhibition Caused by CAPE Treatment

**Figure 6:**
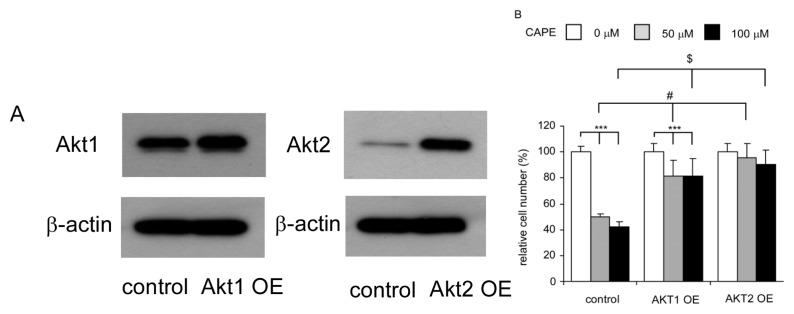
Overexpression of Akt1 and Akt2 in TW2.6 cells rescued inhibition of cell proliferation caused by CAPE treatment. (**A**) Protein expression of Akt1 and Akt2 in TW2.6 cells transfected with empty vector control (control) or TW2.6 cells transient overexpressing Akt1 (Akt1 OE) or Akt2 (Akt2 OE); (**B**) Cellular proliferation of Tw2.6 empty vector control, TW2.6 overexpressing Akt1, and TW2.6 overexpressing Akt2 was assayed by Hoechst dye-based 96-well proliferation assay after being treated with 0, 50, 100 μM CAPE for 24 h. Asterisk *** represents statistically significant difference *p* < 0.001 between the CAPE treatment groups (50 and 100 μM) and the control group (no CAPE treatment) in each TW2.6 cell line. # and $ represents statistically significant difference *p* < 0.001 between the Akt overexpression groups (either Akt1 or Akt2) and the parental TW2.6 cells under treatment of 50 μM CAPE or 100 μM CAPE, respectively. Experiments were repeated three times. Error bars represented standard deviation. The mean and standard deviation represented the average and standard deviation respectively of the results from all 30 wells in the three experiments.

To determine if CAPE suppresses proliferation of TW2.6 cells by inhibiting Akt signaling, we overexpressed Akt1 and Akt2 in TW2.6 cells (Figure 6A). Overexpression of either Akt1 or Akt2 significantly blocked the suppressive effect of CAPE (Figure 6B).

### 2.7. Co-Treatment of CAPE with Chemotherapeutic Drug 5-fluorouracil Suppressed Proliferation of TW2.6 Cells More Efficiently

**Figure 7:**
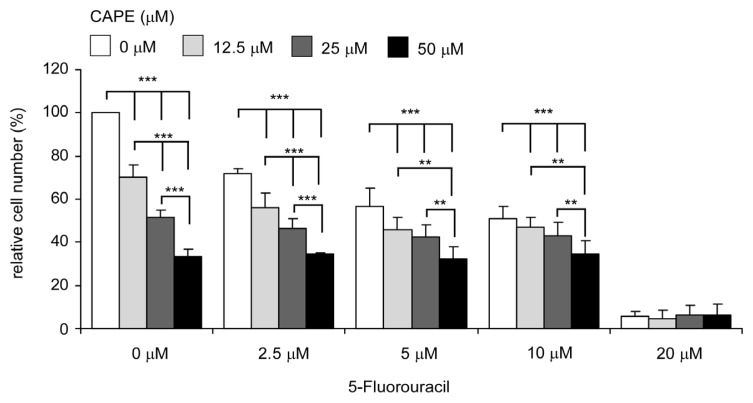
Co-treatment of CAPE and 5-fluorouracil exhibited additive suppression effect on proliferation of TW2.6 cells. TW2.6 cells were treated with increasing concentrations (0, 2.5, 5, 10 and 20 μM) of 5-fluorouracil in the presence of various concentrations (0, 12.5, 25, 50 μM) of CAPE for 48 h. Proliferation of TW2.6 cells was determined by Hoechst dye-based 96-well proliferation assay. Cell number was normalized to control (DMSO treatment only). Experiments were repeated three times. Error bars represented standard deviation. The mean and standard deviation represented the average and standard deviation respectively of the results from all 36 wells in the three experiments. Asterisk (***) represents statistically significant difference (*p* < 0.001) between the treated group and the control group.

We investigated if co-treatment of CAPE with commonly used chemotherapy drug 5-fluorouracil can suppress growth of TW2.6 cells more effectively than 5-fluorouracil treatment alone. IC_50_ of 5-fluorouracil treatment alone was 9.2 μM (Figure 7). Co-treatment of CAPE and 5-fluorouracil exhibited additive suppression effect on proliferation of TW2.6 cells. The IC_50_ of 5-fluorouracil in the presence of 12.5, 25 and 50 μM CAPE was 7.7, 6.7 and 5.0 μM. Therefore, co-treatment with CAPE significantly reduced the dosage of 5-fluorouracil required to suppress the proliferation of TW2.6 oral cancer cells.

## 3. Discussion

Our observations suggested that CAPE treatment suppressed proliferation and colony formation of TW2.6 human oral cancer cells at concentration 5–100 μM. The IC_50_ of CAPE treatment (96 h) was 19.0 μM for TW2.6 cancer cells. Previous study suggested that the achievable concentration of CAPE in human serum is approximately 17 μM [20]. Therefore, oral administration of CAPE is possible to cause regression of oral cancer cells. As the chemotherapy drug 5-fluorouracil for oral cancer is usually given as a topical cream or solution to form a thin coating at skin lesions, CAPE (12.5–50 μM) can be mixed into the 5-fluorouracil cream or solution for oral cancer treatment.

We demonstrated that 50 μM or higher dosage of CAPE is an effective inhibitor of NF-κB activation in TW2.6 cells (Figure 2B). It was not clear why lower dosage (12.5 and 25 μM) of CAPE induced activity of NF-κB in TW2.6 cells (Figure 2B). However, this observation was consistent with our previous report that CAPE treatment at low dosage (10 μM) induced up-regulation of many NF-κB target genes, such as pro-inflammatory cytokines (IFNB1, TNF, and IL8), the matrix metalloproteases (MMP1, MMP2, and MMP9), regulator of morphogenesis and metastasis (TWIST), and cell cycle inhibitor (CDKN1A) [12]. CAPE treatment (50 or 100 μM) suppressed both total abundance and phosphorylation on Serine 536 of NF-κB. Phosphorylation of NF-κB p65 at S536 is required for TNF-α-induced NF-κB activation [21]. NF-κB is an important cell-survival signaling protein. NF-κB plays a key role in regulating cellular response to stress and the immune response to infection [22]. Desregulation of NF-κB has been linked to cancer, inflammation, autoimmune diseases, *etc.* [22]. High expression levels of NF-κB p65 and IKKα was found to correlate to invasiveness, metastasis, and anti-apoptotic activity of OSCC [23]. Therefore, administration of CAPE can be a potential treatment for primary and metastatic OSCC by blocking the NF-κB survival pathway.

**Figure 8:**
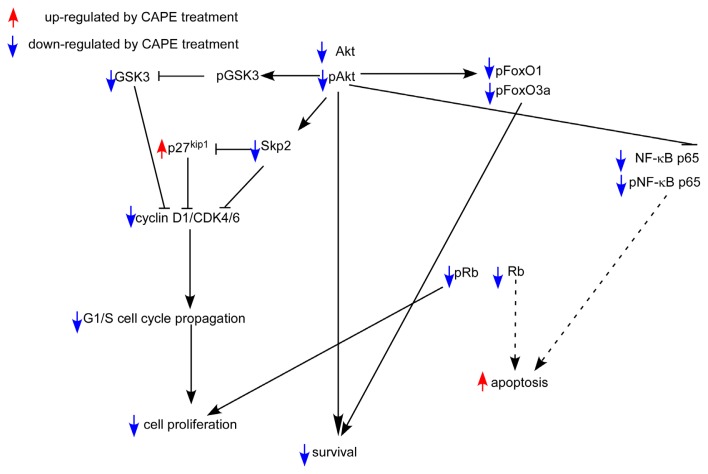
Putative model of anti-cancer effect of CAPE in TW2.6 human oral cancer cells. Protein abundance or activity being stimulated by CAPE treatment are labeled with red upward arrows, while those being suppressed by CAPE treatment are labeled with blue downward arrows. Dash lines indicated possible effects.

Skp2 is a member of the F-box protein family which is responsible for ubiquitination and down-regulation of p27^Kip1^ and other proteins [24,25]. We observed that CAPE treatment decreased Skp2, increased p27^Kip1^, and led to G1 cell cycle arrest. This is consistent with the known function of Skp2 and p27^Kip1^ (Figure 3). Rb is a tumor suppressor protein and is mutated or suppressed in several types of cancers [26]. Reduction in phosphorylation of Rb restricts cell proliferation by inhibiting E2F activity [27]. Cyclin D1 is a protein encoded by CCND1 gene and forms a complex with CDK4 or CDK6. These complexes are essential for cell cycle G1/S transition [28]. Cyclin D1 interacts with Rb and the expression of CCND1 gene is positively regulated by Rb [28]. Akt is a serine/threonine-specific protein kinase activated by phosphatidylinositol 3-kinase (PI3-kinase). Akt plays important role in cell proliferation and survival [29]. There are three mammalian isoforms of this enzyme, Akt1, Akt2, and Akt3 [30,31]. Two phosphorylation sites on Akt, threonine 308 and serine 473, regulate activity of Akt. Phosphorylation of Thr308 on Akt is activated by PDK1 [32]. Phosphorylation of serine 473 is activated by mTOR kinase, its associated protein rector, and SIN1/MIP1 [33,34]. Phosphorylation of these two sites elevates activity of Akt. Reduction of phosphorylation on Ser473 of Akt will decrease the phosphorylation of downstream Gsk-3β Ser9. The reduction in phosphorylation of GSK3β will then increase GSK3β activity [35], which then suppresses the abundance of β-catenin, cyclin D1, and cyclin E [36–38]. FOXO1 is a transcription factor that plays important roles in regulation of gluconeogenesis and glycogenolysis by insulin signaling. Both FOXO1 and FOXO3a can be phosphorylated by Akt [39,40]. FOXO3a is a well known tumor suppressor [41]. Recent studies also suggested that FOXO1 is a tumor suppressor [42]. Phosphorylation of FOXO1 or FOXO3a by Akt will inhibit their activity and resulted in translocation of these proteins out of the nucleus [40]. Down-regulation of FOXO3a activity is frequently observed in several types of cancers [41]. Therefore, decline of phosphorylation of FOXO1 and FOXO3a caused by CAPE treatment will elevate their tumor suppressor activity, which may contribute to the growth inhibition of TW2.6 cells. Down-regulation of Akt, phospho-Akt Ser473, phospho-Akt Thr308, GSK3β, Skp2, phospho-Rb Ser807/811, phospho-FOXO1 Thr24, phospho-FOXO3a Thr 32, and cyclin D1 coupled with increased p27^Kip1^ abundance likely contributed to the induction of G2/M cell cycle arrest and growth inhibition in TW2.6 cells. However, we noticed that protein abundance of total Rb was also suppressed by CAPE treatment (Figure 5). Loss of Rb function will trigger either p53-dependent or p53-independent apoptosis [43]. TW2.6 cells express abundant p53 protein with an A to G mutation at the second base of codon 220 [7]. Decrease of total Rb protein caused by CAPE treatment may contribute to the induction of apoptosis in TW2.6 cells. We summarize the effect of CAPE treatment on different signaling proteins and the potential effect on cell survival, cell cycle, and cell proliferation of TW2.6 cells in Figure 8.

CAPE treatment significantly reduced the protein abundance of Akt1 and Akt2 (Figure 5). Although protein abundance of Akt3 was also suppressed by CAPE treatment, the protein expression levels of Akt1 and Akt2 were more abundant in TW2.6 cells compared to Akt3 (Figure 5). We therefore determined if overexpression of Akt1 or Akt2 may rescue the suppressive effect of CAPE. Overexpression of either Akt1 or Akt2 dramatically blocked the growth inhibition induced by CAPE treatment (Figure 6), confirming that Akt1 and Akt2 are important targets for anticancer function of CAPE in TW2.6 cells.

5-Fluorouracil (also known as 5-FU) is a chemotherapeutic drug for treating different types of cancer. 5-fluorouracil suppresses cancer cells by misincorporating fluoronucleotides into RNA and DNA as well as by inhibiting the nucleotide synthetic enzyme thymidylate synthase [44]. 5-Fluorouracil is widely used for treating advanced head and neck cancer [45]. However, common undesired side effects include diarrhea, nausea, vomiting, mouth sores, poor appetite, watery eyes, photophobia, taste changes, metallic taste in mouth during infusion, and low blood counts (http://chemocare.com/chemotherapy/drug-info/5-fu.aspx). Propolis is a natural medicine used for hundreds of years and is being sold as dietary supplements. CAPE is a pure compound isolated from honeybee hive propolis with no known undesired toxic effects. Our data suggested that co-treatment of CAPE can reduce the dosage required for 5-fluorouracil to suppress proliferation of OSCC cancer cells (Figure 7), which may decrease the uncomfortable syndromes for patients using 5-fluorouracil. Previously, CAPE treatments have been shown to sensitize cancer cells to chemotherapeutic drugs and radiation treatment by inhibiting pathways that lead to treatment resistance in animal models [46]. CAPE treatments have also been shown to protect tissues and organs from chemotherapy-associated toxicities in animal models [14,15,46–54]. Therefore, oral cancer patients receiving chemotherapies may benefit from co-treatment of CAPE, which may enhance the regression of tumors and protect tissues and organs of patients from chemotherapy.

## 4. Material and Methods

### 4.1. Materials

CAPE and 5-fluorouracil were purchased from Sigma (St. Louis, MO, USA).

### 4.2. Cell Culture

TW2.6 cells were maintained in a mixed medium of DMEM (Gibco/Invitrogen, Carlsbad, CA, USA) and Ham’s F12 (Gibco/Invitrogen) medium at 3:1 ratio and supplemented with 10% fetal bovine serum (FBS; Atlas Biologicals, Fort Collins, CO, USA), penicillin (100 U/mL), and streptomycin (100 μg/mL) as suggested [7]. Cells were cultured in incubator at 37 °C, 5% CO_2_, and passaged every 4 days with trypsin.

### 4.3. Hoechst Dye 33258-Based Cell Proliferation Assay

Relative cell number was analyzed by measuring DNA content of cell lysates with the fluorescent dye Hoechst 33258 (Sigma, St. Louis, MO, USA) as described previously [12,13,55–59].

### 4.4. Cell Viability Assay

Cell viability was assessed by MTT (3,4,5-dimethylthiazol-2-yl)-2-5-diphenyltetrazolium bromide) assay [17]. Cells were seeded at a density of 3 × 10^3^ cells per well in a 96-well plate (BD Bioscience). After 24 h, the cells were treated with increasing concentrations of CAPE for 48 h or 96 h. The amount of formazan was determined by measuring the absorbance at 560 nm using a Tecan GENios™ plate reader (Tecan group Ltd, Männedorf, Switzerland) [17]. All results were normalized to the average of the control condition (no CAPE treatment) in each individual experiment. The experiment was repeated three times. Each time ten wells were utilized for each condition. The mean and standard deviation represented the results from all 30 wells in the three experiments.

### 4.5. Soft Agar Colony Formation Assay

TW2.6 cells (8 × 10^3^) were suspended in 0.3% low melting agarose (Lonza, Allendale, NJ, USA) containing mixed medium (DMEM and Ham’s F12 medium at 3:1 ratio and supplemented with 10% FBS) and then layered on top of 3 mL of 0.5% low melting agarose containing mixed medium. Cells were allowed to grow at 37 °C and 5% CO_2_ for 16 days. The plates were stained with 0.005% crystal violet in 30% ethanol for 6 h to detect cell colonies. Number of colonies was counted manually.

### 4.6. Luciferase-Reporter Assay

TW2.6 cells were seeded at 2 × 10^5^ cells/well in a 12-well plate in mixed medium (DMEM and Ham’s F12 medium at 3:1 ratio) containing 10% FBS. 18–24 h after plating, TW2.6 cells were transfected with pRL-TK-Renilla luciferase plasmid (normalization vector; 2.67 ng/well), 4X NF-κB (reporter gene vector; 800 ng/well) using the PolyJet™ *in vitro* DNA transfection reagent (SigmaGen Laboratories, Rockville, MD, USA). 24 h after transfection, cells were treated with increasing concentrations of CAPE. After an additional 24 h, cells were lysed in 100 μL passive lysis buffer (Promega, Madison, WI, USA) and luciferase activity was measured using a Dual-Luciferase kit (Promega) in a 20/20^n^ luminometer Turner Biosystems.

### 4.7. Flow Cytometric Analysis

TW2.6 cells were seeded at a density of 5 × 10^5^ cells in 10-cm dishes in 10 mL of media for 24 h. After additional 48 h of culture in the presence of various concentrations of CAPE, cells were removed with trypsin and fixed in 70% ethanol in PBS overnight at −20 °C. Fixed cells were washed with PBS, treated with 0.1 mg/mL RNase A in PBS for 30 min, and then suspended in 50 μg/mL propidium iodide in PBS. Cell cycle profiles and distributions were determined by flow cytometric analysis of cells using a BD Facscan flow cytometer (BD Biosciences, San Jose, CA, USA). Cell cycle distribution was analyzed using ModFit LT software (Verity Software House, Topsham, ME, USA) as described [12,55–59].

### 4.8. Western Blotting Analysis

Cells were lysed in SDS lysis buffer (240 mM Tris-acetate, 1% SDS, 1% glycerol, 5 mM EDTA pH 8.0) with DTT, protease inhibitors, and a cocktail of phosphatase inhibitors. Antibodies detecting Rb, phospho-Rb Ser807/811, cyclin D1, total Akt, phospho-Akt Ser473, phospho-Akt Thr308, GSK3β, FOXO1, FOXO3a, and phospho-FoxO1 Thr24/phospho-FoxO3a Thr32 were from Cell Signaling (Danvers, MA, USA). Antibodies detecting Skp2, NF-κB (p65), and p27^Kip1^ were from Santa Cruz (Santa Cruz, CA, USA). Antibodies detecting Akt1 and Akt3 were purchased from Millipore (Billerica, MA, USA). Antibodies detecting Akt2 and β-actin were from Novus (Littleton, CO, USA). Antibody for phospho-NF-κB (p65) Ser536 was from Epitomics (Burlingame, CA, USA). Signal of horseradish peroxidase labeled 2nd antibodies was detected by enhanced chemoluminescence reaction (ECL Western Blotting detection kit) (PerkinElmer, Waltham, MA, USA). GAPDH and β-actin were used as loading controls.

### 4.9. Overexpression of Akt1 and Akt2

Cells were seeded at 2.5 × 10^6^ cells/plate in 10 cm dish with DMEM/F12 (3:1) medium containing 10% FBS. After plating for 18 to 24 h, TW2.6 cells were transfected with pCDNA3.1 Vector, Akt1 or Akt2 plasmid, using the PolyJetTM *in vitro* DNA transfection reagent (SigmaGen Laboratories, Rockville, MD, USA). 24 h after transfection, cells were seeded at 3000 cells/well with 100 μL medium in 96-well plates. Cells were then treated with increasing concentrations of CAPE (0, 50 and 100 μM) for additional periods of time (48, 72 and 96 h). Relative cell number was analyzed by measuring DNA content of cell lysates with the fluorescent dye Hoechst 33258 (Sigma) as described previously. Western blotting was used to confirm overexpression of Akt1 and Akt2 proteins.

### 4.10. TUNEL Assay

Cells were grown on cover slides in 24 wells and were treated with 0, 25, 50, 100 μM CAPE for 48 h. Cells were rinsed twice with PBS and subjected to the TUNEL assay using ApoAlert DNA Fragmentation Assay Kit (catalog no. 630108 from Clontech, Mountain View, CA, USA) according to the manufacture's instruction. The TUNEL-stained cells were observed with Olympus confocal microscope at 200 X (FV300, Olympus, Tokyo, Japan).

### 4.11. Data Analysis

Student’s t test (two-tailed, unpaired) was used to evaluate the statistical significance of results from proliferation assay experiments. An Excel add-in program ED50V10 was used for calculating the half maximal inhibition concentration (IC_50_).

## 5. Conclusions

Our observations suggested that CAPE administration may be a potential adjuvant therapy for OSCC oral cancer patients. CAPE suppressed cell proliferation of TW2.6 oral cancer cells via inhibition of Akt signaling. Oral cancer patients receiving chemotherapy of 5-fluorouracil may benefit from co-treatment of CAPE, which may enhance the regression of tumors and reduce the required dosage of 5-fluorouracil.
